# Bona Fide Intersegmental Plane Hepatectomy Along the Glissonean Branches (BIPHG)

**DOI:** 10.1002/ags3.70180

**Published:** 2026-01-20

**Authors:** Go Wakabayashi, Taiga Wakabayashi

**Affiliations:** ^1^ Center for Advanced Treatment of Hepatobiliary and Pancreatic Diseases, Ageo Central General Hospital Saitama Japan

**Keywords:** anatomical liver resection, laparoscopic liver resection, liver cancer, minimally invasive liver resection, robotic liver resection

## Abstract

This review traces the author's personal journey over three decades in the evolution of liver resection techniques, culminating in the proposal of the *Bona Fide Intersegmental Plane Hepatectomy Along Glissonean Branches* (BIPHG). Beginning with pioneering experiences in laparoscopic and robotic liver surgeries, the author highlights key anatomical insights—particularly Laennec's capsule and intersegmental planes—that have advanced surgical precision. Transitioning from laparoscopy‐assisted to pure laparoscopic donor hepatectomy illustrated the importance of safety, visualization, and anatomy‐guided dissection. Inspired by innovations in intraoperative imaging and microanatomy, the author introduces BIPHG—an innovative tension‐based technique utilizing indocyanine green (ICG) fluorescence to define natural intersegmental boundaries along Glissonean branches. This method emphasizes real‐time visualization, enabling accurate, minimally invasive, and function‐preserving liver resections. The review underscores the significance of standardizing definitions and assessment tools, such as the difficulty score, to facilitate broader adoption. Looking forward, ongoing technological and anatomical advances are expected to further refine these approaches, positioning BIPHG as a promising, safe, and precise strategy in minimally invasive liver surgery. This personal narrative aims to contribute to the continued evolution of anatomy‐based, minimally invasive hepatic resection techniques.

## Abstract 2

This review proposes *Bona Fide Intersegmental Plane Hepatectomy along the Glissonean Branches* (BIPHG), a minimally invasive, anatomy‐based liver transection technique using Glissonean control and ICG fluorescence to identify the true intersegmental plane. Controlled tension—especially in robotic surgery—enables precise, efficient, and parenchyma‐sparing anatomical liver resection, supporting safe and reproducible minimally invasive practice.

## 1 My Encounter with Laparoscopic and Robotic Liver Resection

Thirty years ago, in July 1995 at Keio University Hospital, I performed my first laparoscopic partial liver resection for a solitary metastasis [1]. This was after a case where an opera singer, wanting to preserve her ability to sing, requested a laparoscopic approach following a colectomy for colon cancer. Reflecting on these advances, I am amazed at how far we've come [2].

In the early cases—about two dozen—concerns about bleeding during parenchymal transection and ergonomic challenges were common due to limited experience. In March 2000, I performed Asia's first Da Vinci robotic cholecystectomy at Keio [3]. While initially seeing little benefit for gallbladder surgery, I believed robotic techniques would excel in liver resections. In July 2001, I performed Keio's first robotic liver resection, which was also the world's first according to Intuitive Surgical [4]. At that time, only a bipolar Maryland dissector was available, limiting the scope. It was not until 21 years later that robotic liver resection was covered by insurance in Japan, marking a significant milestone.

## 2 From Laparoscopy‐Assisted to Pure Laparoscopic Donor Hepatectomy

Since Keio University Hospital launched its living donor liver transplantation program in April 1995 [5], we have aimed to reduce the physical and emotional burdens on healthy donors through minimally invasive techniques. After Cherqui and Soubrane's 2000 report of a fully laparoscopic left lateral sector donor hepatectomy [6], we began exploring similar approaches, initially combining laparoscopy‐assisted resections with a midline upper abdominal incision for safety. After successful pig model simulations, we performed this procedure in February 2004, with donors appreciating that scars wouldn't affect future pregnancies [1]. At Iwate Medical University, we started living donor transplants in January 2007 and adopted laparoscopy‐assisted donor hepatectomy from August of that year [7]. Emphasizing safety, we used a midline incision with a hanging maneuver to facilitate precise transection, even in donors with deep thoracic cavities, maintaining vascular control with standard clamps and direct visualization [8].

However, in April 2012, a tragic intraoperative death occurred at Lahey Clinic due to uncontrollable bleeding during hand‐assisted donor hepatectomy [9]. Although such procedures are sometimes categorized as hybrid, the obstruction of the field of view inherent in hand‐assisted techniques led us to avoid their use for donor surgery [10]. This event prompted us to reconsider; for larger donors with deep thoracic cages, we hypothesized that a pure laparoscopic approach could improve safety and visualization. Inspired by Soubrane's presentation of a pure laparoscopic right donor hepatectomy at the 2012 IHPBA Congress, we began performing pure laparoscopic left donor hepatectomies at Iwate in December 2012 [11]. This marked a significant step forward in advancing minimally invasive donor procedures.

Before retiring in 2015, I performed 40 laparoscopy‐assisted and 14 pure laparoscopic donor hepatectomies. A retrospective analysis confirmed the safety and feasibility of the pure laparoscopic method, with blood loss about one‐third of that in LADH [12]. These results serve as a proof‐of‐concept, supported by a pig model study on intraoperative bleeding under pneumoperitoneal pressure [13].

*Throughout every step of surgery, each maneuver should serve as preparation for the next*.

This principle, rooted in my experience with living donor transplants, emphasizes imagining worst‐case scenarios and planning accordingly, which has been crucial for donor safety and intraoperative decision‐making.

## 3 Significance of the Morioka Consensus Conference

Recent advances in endoscopic liver surgery have shown benefits like magnification and reduced venous bleeding due to pneumoperitoneum, allowing for more precise parenchymal transection [14]. These developments support the idea that laparoscopic liver resection (LLR) may be better than open hepatectomy [15]. To promote this worldwide, the Morioka Consensus Conference was held, addressing 17 key questions: the first seven about the benefits and risks of LLR, and the remaining 10 about technical aspects [16]. Initially, we reviewed existing literature [17], but because of the lack of randomized controlled trials and limited evidence, we adopted an independent, jury‐based consensus approach [18]. Technical recommendations were refined based on literature and expert opinions. A systematic review was conducted for each clinical question [19], resulting in the publication of 25 relevant studies after the conference [20]. This collaborative effort has greatly advanced minimally invasive liver surgery [21].

Two main points emerged from the discussions:

1. *The development of a difficulty score* to assess surgeon expertise for new procedures was published before the conference [22], this score was updated during the meeting [20] and has been validated in subsequent studies [23, 24, 25]. It is now widely used to evaluate the complexity of laparoscopic liver resections and has been adapted for other minimally invasive procedures, such as laparoscopic pancreatectomy [26] and robotic liver resection [27].
2. *The Glissonean approach* [28] and the importance of anatomical liver resection (AR). Sugioka's gate theory [29] highlighted using the Glissonean technique by experienced surgeons. While the benefits of AR are debated, many support it, following Couinaud's regional classification based on portal and hepatic veins [30]. Further discussion on implementing AR in practice continues.

## 4 Clinical Outcomes of Anatomical Liver Resection

Looking across tumor types, AR remains central to the discussion of oncologic strategy. For hepatocellular carcinoma (HCC), a series of landmark Japanese studies consistently demonstrated that AR reduces local recurrence and improves long‐term outcomes compared with non‐anatomical resection (NAR) [31, 32, 33, 34]. A RCT further confirmed that AR significantly lowered 2‐year local recurrence and delayed the timing of the first recurrence, although overall survival was comparable between groups [35]. More recently, an international multicenter study showed that AR improved 5‐year recurrence‐free survival (63.9% vs. 52.0%) and reduced non‐transplantable recurrences, particularly in patients with intermediate tumor burden [36]. Conversely, a multi‐institutional Japanese analysis of HCC with microportal invasion (vp1) found no survival advantage for AR after propensity score adjustment, though local recurrence was lower [37]. Taken together, these findings suggest that the benefit of AR depends not only on resection extent but also on underlying tumor biology and hepatic reserve.

Clinicopathological studies have provided further biological rationale for these clinical observations. Shindoh et al. demonstrated that complete removal of the tumor‐bearing portal territory reduces local recurrence and improves disease‐specific survival [38], while Cho et al. reported that postoperative remnant‐liver ischemia was associated with early recurrence and poor survival [39]. These findings support the pathophysiological basis for resection along true intersegmental planes and Glissonean pedicles, eliminating microscopic disease spread and minimizing ischemic injury.

Recent developments in parenchyma‐sparing yet anatomically precise procedures—particularly those guided by Glissonean approaches and ICG fluorescence—represent a logical evolution of this concept. Our own single‐center analysis showed that laparoscopic limited anatomical resections (LAR) can achieve favorable long‐term outcomes for both HCC and colorectal liver metastases (CRLM) [40].

For CRLM, parenchymal‐sparing resection has been widely adopted as standard practice, providing comparable survival, better perioperative recovery, and facilitating repeat hepatectomy when needed [41]. However, in biologically aggressive subsets such as KRAS‐mutated CRLM, portal‐territory‐oriented AR has been associated with improved disease‐free survival [42]. Thus, while parenchymal preservation remains the prevailing trend, AR guided by biological aggressiveness should still be considered for maximizing oncologic control.

In Japan, where liver transplantation remains limited and resection continues to serve as the main curative option, our focus has naturally shifted toward how AR can be performed—refining its precision, safety, and reproducibility through continuous surgical innovation.

## 5 Intersegmental Plane Liver Parenchymal Transection

**FIGURE 1:**
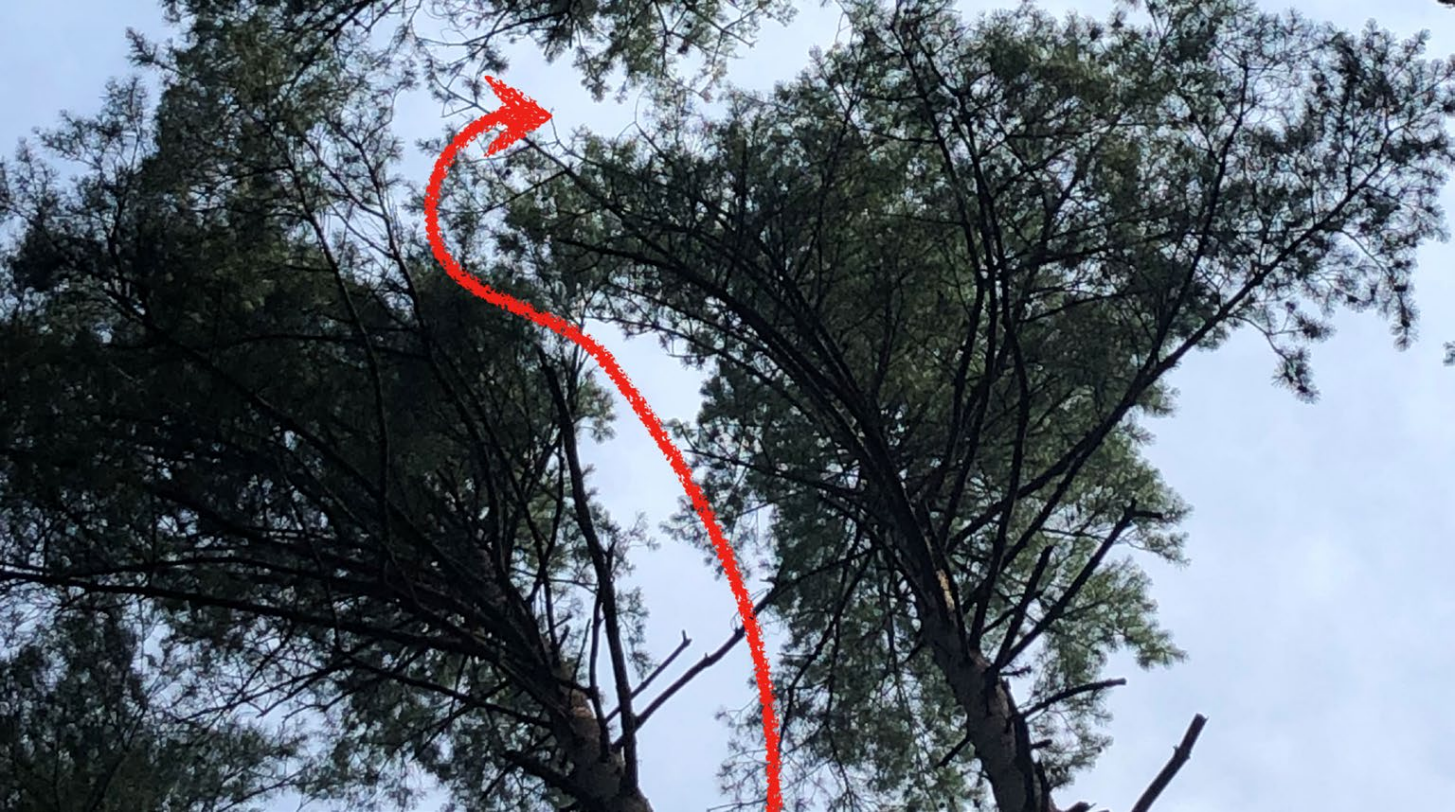
Ideal liver resection: Anatomical dissection along Glissonean branching patterns.

Our first laparoscopic anatomical liver resection—an approach less extensive than a sectionectomy—involved a medial sectionectomy combined with ventral anterior sectionectomy for hepatocellular carcinoma [43]. For this centrally located tumor, we employed a Glissonean approach, targeting several Glissonean branches extending ventrally within the liver parenchyma, and resected the discolored area visible on the liver surface. Since this procedure was performed in 2011, indocyanine green (ICG) imaging was not available laparoscopically [44]. Given the patient's cirrhosis and compromised hepatic reserve, we opted for an intraparenchymal Glissonean approach, aiming to preserve as much healthy liver tissue as possible while ensuring adequate resection margins for the tumor. Around that time, I began to consider that directing liver resections along Glissonean branches was a logical strategy. Liver resection should be performed along the Glissonean branches because a gap exists between them, resembling the branching pattern of a tree (Figure 1).

**FIGURE 2:**
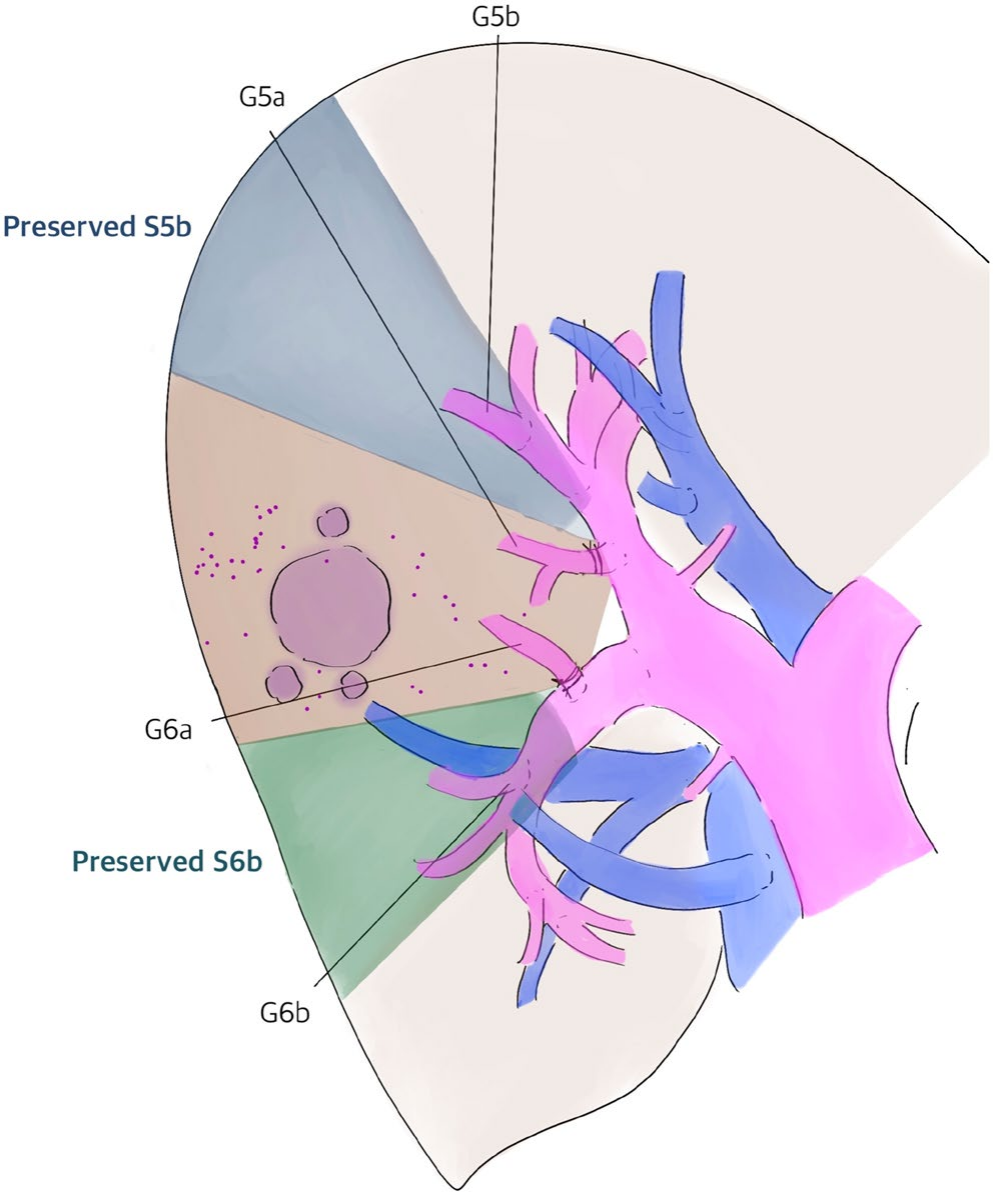
Subsegmentectomies of S5 and S6 (H5^S^6^S^) for hepatocellular carcinoma. Parenchyma‐sparing anatomical resection combined with subsegmentectomy for optimal tumor margin securing.

The first case of LAR using ICG is schematically illustrated in Figure 2. The hepatocellular carcinoma is situated between S5 and S6, as tumors can grow unpredictably. To ensure complete tumor removal, we opted to excise one Glissonean pedicle in S5 and another in S6. This technique was later defined as subsegmentectomies of S5 and S6 (H5^S^6^S^). Approximately 1–2 cm of liver parenchyma was dissected from the hilum, after which the Glissonean pedicles in S5 and S6 were carefully isolated and secured with bulldog clamps. Subsequently, 0.5 mg/body of indocyanine green (ICG) was administered intravenously, clearly visualizing the demarcation line on the liver surface. This technique has been used for parenchymal‐sparing LAR at Ageo Central General Hospital (ACGH) [45], with results published elsewhere [40].

**FIGURE 3:**
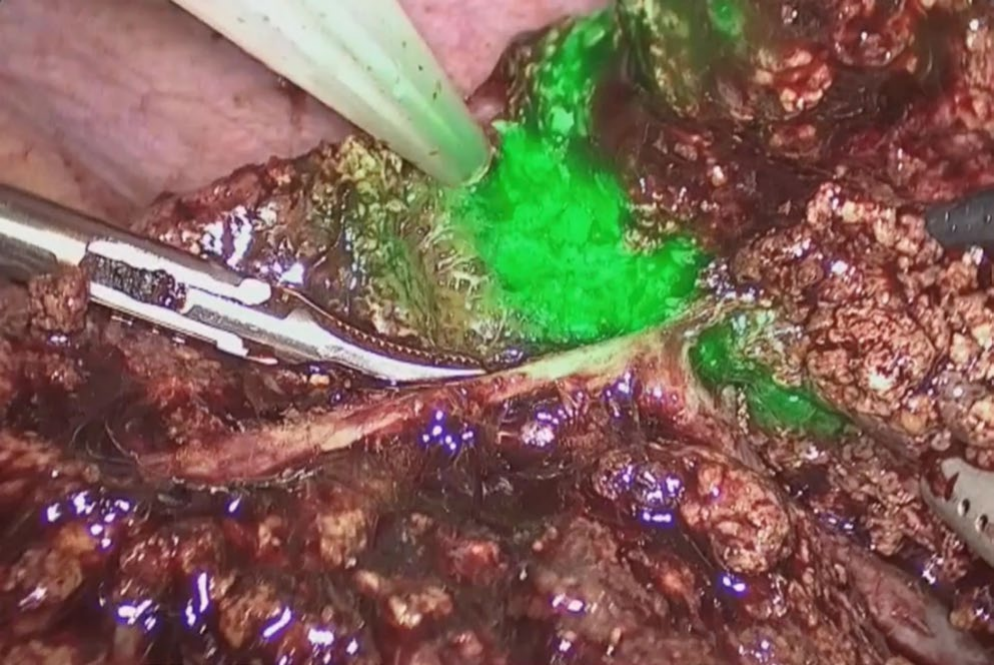
Intersegmental plane between S8 ventral and S8 dorsal visualized by ICG negative staining. Anterior fissure vein along the plane with a crossing vein.

I vividly remember the shock I felt when I first visualized the intersegmental plane using ICG (Figure 3). During subsequent resections from the hilum employing the Glissonean approach and observing ICG‐negative staining, I realized that the portal tertiary border identified by ICG did not contain the thick Glissonean branch, only the hepatic veins. The border of the cone unit, as proposed by Takasaki [28], is the intersegmental plane, and combining these planes to perform an AR offers a procedure that is both effective and function‐preserving. At the PAM Consensus Conference [46], held online in February 2021, we defined monosegmentectomy and subsegmentectomy, incorporating the concept of the Glissonean approach (which was absent in the Brisbane 2000 terminology). These definitions were subsequently published as part of the Tokyo 2020 consensus terminology [47]. We hope this will stimulate further research into limited, parenchyma‐sparing AR and promote their standardization as minimally invasive surgical techniques.

## 6 Advantages of Resection Along the Intersegmental Plane

The key points are detailed below:

1. *Anatomical simplicity*: The intersegmental plane connects peripheral branches of Glisson, with the hepatic vein being the only significant structure in this zone [48]. Parenchymal transection along this plane can be performed with bipolar vessel sealers, which are used to close and divide crossing hepatic veins—eliminating the need for clips. The concept of the “chicken claw,” as described by Itano et al. [49], provides a sound understanding of this plane's anatomy and serves as a helpful guide.
2. *Efficiency and speed*: Because the intersegmental plane involves fewer structures—even in livers with significant fibrosis—transection is faster and easier. Using a cavitron ultrasonic surgical aspirator (CUSA) and the back‐scoring technique proposed by Honda et al. [50], which transcribes the plane without Glissonean branches, increases procedural efficiency.
3. *Alignment with Glissonean bifurcation*: When liver resection follows Glissonean bifurcations from the hilum, it naturally aligns with the intersegmental plane. The first Glissonean branch corresponds to hemi‐hepatectomy, the second to sectionectomy, and the third to subsegmentectomy [51]. Sugioka's gate theory [29] applies to resection up to the second branch—where the Laennec capsule resides—but from the third branch onward, intrahepatic Glissonean approaches are employed, often involving parenchymal destruction. Despite these differences, planned resections along the intersegmental plane are compatible with a Glissonean approach.

## 7 True Intersegmental Plane Liver Resection Along the Glisson's Branches

Recent publications have addressed key challenges associated with parenchymal transection in robotic liver resections, particularly focusing on overcoming limitations related to the use of CUSA. In robotic procedures, the use of CUSA requires the surgeon to operate it manually, often necessitating a third robotic arm for field preparation. Unlike laparoscopic approaches, this setup results in the loss of one forceps, complicating tissue manipulation. Without CUSA, liver transection is performed by applying tension to both transected surfaces, utilizing a double bipolar technique to facilitate tissue dissection.

Abe et al. [52] reported a technique involving gentle stroking of the liver parenchyma with Maryland forceps to gradually separate tissue planes. Similarly, Nitta and colleagues [53] employed four clamp crush techniques with Maryland forceps to facilitate transection. Recently, we introduced a comparable approach in robotic liver resection by applying tension to the transected surface. We attach a fishing line to the liver parenchyma using a 3‐0 Vicryl suture, then prepare the operative field with significant tension maintained via an Endo Close (Medtronic Japan, Tokyo). As the transection progresses, the tension required to open the surface is gradually increased through the third robotic arm. Subsequently, by gently mobilizing the Maryland forceps craniodorsally along the intersegmental plane—identified using indocyanine green (ICG) fluorescence imaging—parenchymal dissection advances, reminiscent of Moses parting the Red Sea (Videos S1 and S2).

This technique was demonstrated in a video presentation at the ILLS (International Laparoscopic Liver Society) Conference in Seoul, June 2025. We described this phenomenon using the term “tearing,” a description echoed by Kim et al. [54], who observed a similar “detachment” phenomenon. To our knowledge, this represents the first report of a true intersegmental liver resection performed along the Glissonean branches using ICG fluorescence, employing relatively strong tension to the transected surface. While an official nomenclature awaits consensus, we have termed it the “Bona Fide Intersegmental Plane along Glissonean Branches (BIPHG)”—an efficient, rational, and innovative approach to liver parenchymal transection.

While BIPHG represents a progression beyond conventional intersegmental plane hepatectomy, several significant differences between the two approaches warrant careful consideration. The application of tension on the hepatectomy plane improves the delineation of the true intersegmental plane, thereby facilitating liver parenchymal transection with minimal force. During active liver parenchymal transection with CUSA, intersegmental plane hepatectomy can be effectively achieved by using the hepatic vein as a landmark, thereby reducing dependence on ICG segmentation or Glissonian branches. To clarify these distinctions, a comparative table is presented to underscore the significance of each factor (Table 1).

**TABLE 1:** Comparison between BIPHG and conventional intersegmental plane hepatectomy.

	BIPHG	Conventional intersegmental plane hepatectomy
Tension to the resection line	+++	+
ICG segmentation	+++	++
Chicken's claw	+++	+++
Glissonean approach	+++	++
Double bipolar method	++	++
CUSA	++	+++

## 8 Requirements for Bona Fide Intersegmental Plane Hepatectomy Along Glissonean Branches (BIPHG)

**FIGURE 4:**
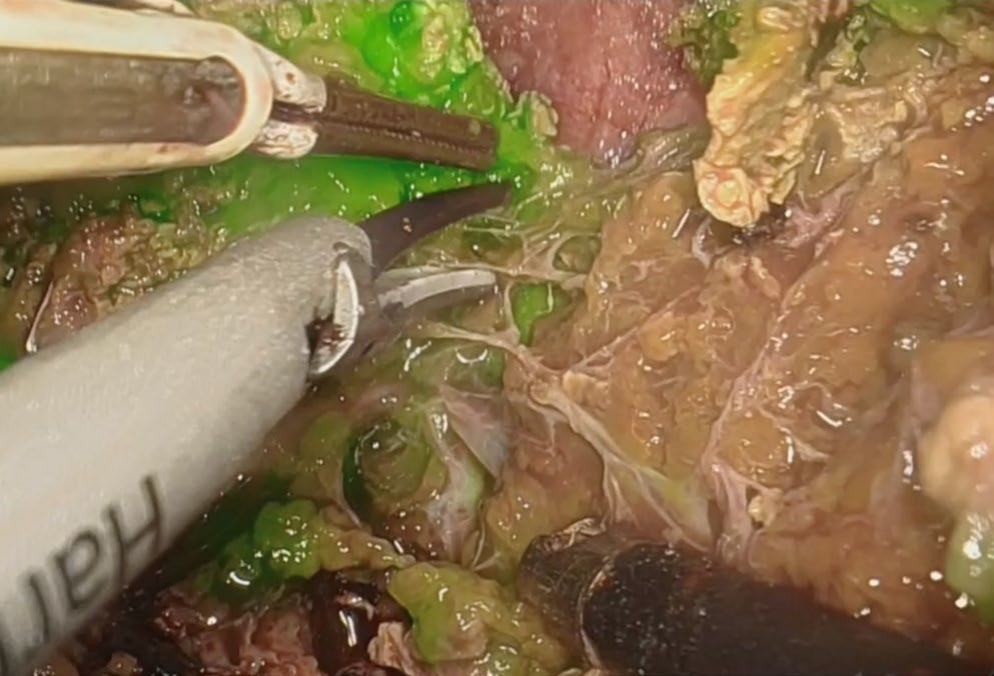
“Chicken claw” structures located at the border between S4 and S8, identified by ICG‐negative staining.

ICG staining is crucial for accurately outlining the intersegmental plane. In sectionectomies or larger resections, a relatively straight craniocaudal line often appears on the surface, allowing intersectional hepatectomy even without ICG. For hemi‐hepatectomies, the plane follows the Cantlie line, facilitating dissection along the middle hepatic vein with or without ICG guidance. Intersegmental hepatectomy can follow the “chicken's claw” boundary, as proposed by Itano et al. [49]. ICG staining enhances boundary visibility, especially for segments oriented perpendicular to the body axis, such as between S2 and S3, S5 and S8, and S6 and S7. The chicken's claw at the border of S4 and S8, distinguished by ICG‐negative staining, is shown in Figure 4. Although small hepatic veins along these borders may be visible, exposing them can be more difficult than visualizing main veins.

In ICG‐positive staining, the transection surface indicates a portal vein gap; in ICG‐negative staining, it shows a combined gap of portal vein, hepatic artery, and bile duct walls supplied by arterial flow. We are currently conducting a randomized trial at ACGH comparing ICG‐positive and negative staining during segmentectomy and sectionectomy [55]. Preliminary results suggest negative staining provides a more consistent, reproducible boundary, with outcomes expected within 2–3 years to determine if there is a significant difference.

**FIGURE 5:**
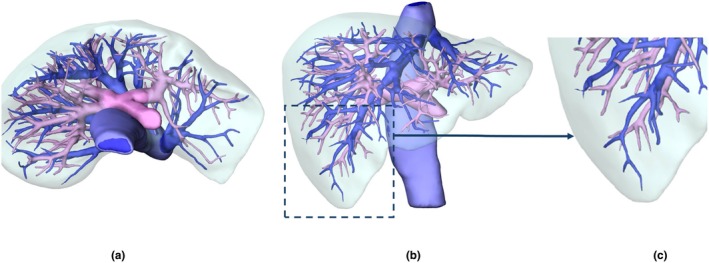
Direction of hepatic veins and Glissonean branches. (a) From dorsal to ventral (caudal view). (b) From cranial to caudal (ventral view). (c) Parallel arrangement in the periphery creates turf grain.

The next step is to apply tension to both sides of the transected liver surface. In robotic hepatectomy—often with one arm missing—a 3‐0 Vicryl suture is placed on the liver parenchyma and pulled outward to retract the surface. Robotic manipulation, such as grasping a sponge for suction, generates significant tension. Even slight contact with the bona fide intersegmental plane, visualized via ICG staining, can produce a sensation of liver parenchymal splitting. Abe et al.'s “trac & pac” technique effectively captures this sensation. Even in laparoscopic hepatectomy, following the CUSA along the plane quickly exposes the transection zone with minimal resistance. Robotic hepatectomy accentuates this effect, as the surface is mobilized under greater tension. During transection along the intersegmental plane, a distinctive “turf grain” pattern appears, running either with (prograde) or opposite (retrograde) to the Glissonean and hepatic vein branches. From the hilum, the Glissonean branches fan dorsoventrally, with the grain running dorsoventrally (Figure 5a). From the hepatic vein root, veins fan cranially to caudally, with the grain running craniocaudally (Figure 5b). Honda et al. [50] observed that these turf grains generally align with the vascular structures, running either dorsoventrally or craniodorsally.

Sugioka's concept of Laennec's capsule suggests it surrounds both Glissonean branches and hepatic veins within the intersegmental plane [56]. These structures form a web‐like network, with peripheral branches and veins interconnected by Laennec's capsule. The peripheral Glissonean branches face each other across the true intersegmental plane, with associated peripheral hepatic veins running alongside them. Larger hepatic veins course along or cross this plane, but Glissonean branches rarely do. Dividing the liver along the plane where subsegmental branches enter reduces crossing hepatic veins. Essentially, all peripheral Glissonean branches and veins run parallel (Figure 5c), connected by Laennec's capsule, which may form a web—possibly the Laennec web—linking these structures (personal communication with Prof. Sugioka). Although not yet definitively proven, many Laennec webs are likely present within the true intersegmental plane, with variations in density and tensile strength among patients that influence the force required for precise transection. Therefore, the key to successfully entering the bona fide intersegmental plane is to apply tension to the dissected surface and split along the boundary revealed by ICG staining in the direction of the grain. This method optimizes the dissection and ensures precise traversal of the intersegmental plane.

## 9 BIPHG Arrived Through Advances in Minimally Invasive Liver Resection

Controlling bleeding during liver transection has been a major challenge for over 40 years. Recent advances in minimally invasive liver resection [57] have culminated in the development of Bona Fide Intersegmental Plane Hepatectomy Along Glissonean Branches (BIPHG), with several key points:

- *Pneumoperitoneum* reduces bleeding and facilitates exposure of hepatic veins [15]. Innovations in energy devices—such as bipolar hemostatic forceps and monopolar coagulation—improve vessel coagulation within the intersegmental plane.
- *Anatomical landmarks*, including Glissonean branches and hepatic veins, are used for standard caudal approach resections, confining bleeding primarily to hepatic veins, which can be effectively controlled with agents like SURGICEL and SURGIFLO.
- *Enhanced visualization* through magnifying endoscopy deepens understanding of microanatomy, such as Laennec's capsule and the “chicken claw” structures. Systematization of anatomy, including the “four landmarks” and “six gates,” improves reproducibility and safety [29]. Furthermore, the “chicken‐claw” technique minimizes unnecessary resection of Glissonean branches during parenchymal transection [49]. This facilitates intersegmental plane hepatectomy while preserving blood flow to the residual liver.
- *ICG fluorescence imaging* provides precise boundary visualization by coloring portal vein territories, enabling accurate monosegmentectomy and subsegmentectomy that were previously difficult [45]. Advances in infrared (IR) camera tech allow real‐time overlay guidance, greatly enhancing safety and precision.
- *Robotic hepatectomy* simplifies the Glissonean approach with multi‐articulated forceps and increases tension on the transection plane. As previously mentioned, intersegmental plane hepatectomy—initially feasible with the use of ICG during laparoscopic procedures—has evolved and advanced into BIPHG with the adoption of robotic hepatectomy [58].

## 10 Conclusion and Future Outlook

The development of liver resection techniques over the past 40 years has been profoundly transformative, marked by groundbreaking innovations such as brain‐dead donor liver transplantation, living donor liver transplantation, laparoscopic liver resection, and robotic liver resection. I have been privileged to witness and actively participate in these remarkable advancements firsthand. These innovations are interconnected. As surgical techniques have been refined, our understanding of liver anatomy—particularly microanatomy—has deepened. I anticipate that liver resection methods will continue to evolve, moving toward more precise, anatomical approaches for all cases.

In the past, procedures like gastrectomy and colon resection were often performed as partial resections; however, the recognition that AR along vascular structures offers greater rationality has shifted the paradigm. Nowadays, oncological surgeries increasingly focus on anatomically appropriate resections, including regional lymph node dissection. Because the liver is a solid organ with indistinct surface landmarks, many resections are performed as partial surface excisions. However, with the widespread adoption of techniques like BIPHG, we aim to perform LAR even for superficial liver cancers, ensuring that areas of ischemia or congestion in the residual liver are minimized or avoided.

Furthermore, BIPHG provides a strong rationale for the implementation of robotic liver resection. Its widespread adoption is expected to enhance the efficiency and safety of both robotic donor liver resections and robotic anatomical liver resections [59].

Looking ahead, continued advances in understanding liver microanatomy and surgical technology will undoubtedly lead to safer, more effective, and more precise liver resections, ultimately improving patient outcomes.

## Author Contributions

**Go Wakabayashi:** conceptualization, writing – original draft, writing – review and editing, formal analysis, visualization, methodology, data curation. **Taiga Wakabayashi:** conceptualization, writing – original draft, writing – review and editing, formal analysis, visualization, methodology, data curation.

## Funding

The authors have nothing to report.

## Ethics Statement

The authors have nothing to report.

## Conflicts of Interest

The authors declare no conflicts of interest.

## Supporting information


**Video S1:** Bona fide right anterior sectionectomy and right hepatic vein resection (H58‐RHV) for intrahepatic cholangiocarcinoma.

**Video S2:** Bona fide right hepatectomy for hepatocellular carcinoma in a cirrhotic patient.
